# Agreement between frozen section and histopathology to detect malignancy in adnexal masses according to size and morphology by ultrasound

**DOI:** 10.61622/rbgo/2024rbgo63

**Published:** 2024-07-26

**Authors:** Clarissa de Andrade Amaral, Priscila Grecca Pedrão, Luani Rezende Godoy, Yasmin Medeiros Guimarães, Cassia Arantes Petroni Macedo, Marcia Appel, Guilherme Spagna Accorsi, Jeferson Rodrigo Zanon, Ricardo dos Reis

**Affiliations:** 1 Hospital de Clínicas de Porto Alegre Universidade Federal do Rio Grande do Sul Porto Alegre RS Brazil Hospital de Clínicas de Porto Alegre, Universidade Federal do Rio Grande do Sul, Porto Alegre, RS, Brazil.; 2 Hospital de Amor Barretos SP Brazil Hospital de Amor, Barretos, SP, Brazil.; 3 Faculdade de Ciências da Saúde de Barretos Dr. Paulo Prata Barretos SP Brazil Faculdade de Ciências da Saúde de Barretos Dr. Paulo Prata, Barretos, SP, Brazil.; 4 Faculdade de Medicina de Catanduva Catanduva SP Brazil Faculdade de Medicina de Catanduva, Catanduva, SP, Brazil.

**Keywords:** Adnexal diseases, Frozen section procedure, Histopathology, Ovarian neoplasms, Ultrasonography

## Abstract

**Objective:**

Management of suspect adnexal masses involves surgery to define the best treatment. Diagnostic choices include a two-stage procedure for histopathology examination (HPE) or intraoperative histological analysis – intraoperative frozen section (IFS) and formalin-fixed and paraffin-soaked tissues (FFPE). Preoperative assessment with ultrasound may also be useful to predict malignancy. We aimed at determining the accuracy of IFS to evaluate adnexal masses stratified by size and morphology having HPE as the diagnostic gold standard.

**Methods:**

A retrospective chart review of 302 patients undergoing IFS of adnexal masses at Hospital de Clínicas de Porto Alegre, between January2005 and September2011 was performed. Data were collected regarding sonographic size (≤10cm or >10cm), characteristics of the lesion, and diagnosis established in IFS and HPE. Eight groups were studied: unilocular lesions; septated/cystic lesions; heterogeneous (solid/cystic) lesions; and solid lesions, divided in two main groups according to the size of lesion, ≤10cm or >10cm. Kappa agreement between IFS and HPE was calculated for each group.

**Results:**

Overall agreement between IFS and HPE was 96.1% for benign tumors, 96.1% for malignant tumors, and 73.3% for borderline tumors. Considering the combination of tumor size and morphology, 100% agreement between IFS and HPE was recorded for unilocular and septated tumors ≤10cm and for solid tumors.

**Conclusion:**

Stratification of adnexal masses according to size and morphology is a good method for preoperative assessment. We should wait for final HPE for staging decision, regardless of IFS results, in heterogeneous adnexal tumors of any size, solid tumors ≤10cm, and all non-solid tumors >10cm.

## Introduction

Management of adnexal masses is still challenging because of the need to remove the tumor to define the type of treatment for affected women.^(1)^Diagnostic choices include a two-stage procedure for collection of tissue samples for formalin fixation and paraffin embedding, or intraoperative histological analysis, known as intraoperative frozen section (IFS) procedure.^(2-5)^IFS would ideally prevent both overtreatment and the need for additional surgical procedures; however, a variety of factors have been implicated in the diagnostic failure of IFS, including size, histologic type, and clinical and sonographic characteristics of the lesion, patient age and menopausal status, and pathologist experience.^(2,6)^ The usefulness of IFS is especially questionable or limited for borderline tumors.^(7-9)^

Prior to treatment, preoperative assessment also plays a crucial role in the management of adnexal masses. A recent meta-analysis, regarding the diagnostic accuracy of different pre-operative ultrasound methods for differentiating benign from malignant adnexal masses, has shown better diagnostic accuracy of simple ultrasound-based rules as compared to risk malignancy index to characterize ovarian pathology.^(8)^In addition, Rogers et al. (2014)^(10)^ have shown that “mass size of ≥ 8 cm was 19 times more likely to identify a malignant adnexal mass over a benign case by odds ratio” (p. 121) in pre-adolescents (12 years of age).^(10)^Those authors also state that a combination of tumor complexity and size was more accurate to identify malignancies than each of these criteria individually.

Taking these aspects into consideration, the present study was developed to determine the accuracy of IFS to evaluate adnexal masses stratified by size and morphology, having histopathology examination (HPE) as the diagnostic gold standard. With that, we expected to identify, with ultrasound, a subgroup of patients in whom we need to wait for final HPE for staging decision despite a negative frozen section.

## Methods

Informed consent was not required due to the retrospective nature of the study. A cross-sectional study based on a retrospective chart review was conducted. All 302 patients who underwent IFS of adnexal masses at Hospital de Clínicas de Porto Alegre (HCPA), Brazil, between January 2005 and September 2011 were studied. The following data were collected from patient charts: birth date; date of first visit; menopausal status; parity; symptoms (lower abdominal pain); past medical or family history of breast neoplasms, ovarian neoplasms, or malignant syndromes; past surgical history; relevant physical examination findings (presence or absence of palpable mass, mass smooth or irregular, infraumbilical or both infra and supraumbilical), preoperative serum CA-125 levels (classified as <35 IU/mL or ≥35 IU/mL), surgical approach (laparoscopic or laparotomy), intraoperative capsule rupture, sonographic size (≤10 cm or >10 cm)^(10)^and characteristics of the lesion (presence of solid component; presence and type of septation, vegetations, or calcifications; presence of ascites; presence of peritoneal implants), and diagnosis established in IFS and HPE.

Staff pathologists at the Hospital de Clínicas de Porto Alegre Pathology Department performed IFS examinations on all adnexal masses treated surgically at the hospital, followed by conventional HPE for a definitive diagnosis. For the purposes of this study, the following data were collected from IFS and HPE reports: histological type, grade, and size of the lesion. The pathologists who made the final diagnosis were not aware of the results of the frozen section examination. In this study, there was a pathologist for the IFS and another for FFPE.

For assessment of the agreement between IFS and HPE for diagnosis of lesions as benign, borderline, or malignant, cases were divided into eight groups according to sonographic size and morphology of the adnexal mass. Groups 1 to 4 included lesions ≤10 cm in size: unilocular (homogeneous) lesions in group 1; septated cystic lesions in group 2; heterogeneous (solid/cystic) lesions in group 3; and solid lesions in group 4. Groups 5 to 8 included lesions >10 cm in size: 5, unilocular (homogeneous) lesions in group 5, septated cystic lesions in group 6; heterogeneous (solid/cystic) lesions in group 7; and solid lesions in group 8.

Quantitative variables, such as age and parity, were expressed as means and standard deviations depending on the normality of distribution. Qualitative variables, such as symptoms, family history of malignancy, surgical history, tumor characteristics, surgical approach, and occurrence of intraoperative capsule rupture were described as present or absent. Statistical analysis consisted of the chi-square test for between-group comparisons of categorical variables. When the chi-square test was not applicable, Fisher’s exact test was used to calculate the likelihood of data distribution. The kappa statistic for agreement between IFS and HPE was calculated for each group.^(11)^Data were analyzed in the Statistical Package for the Social Sciences (SPSS) 18.0 software package. Statistical significance was set at p < 0.05.

The present study was approved by the Research Ethics Committee at Hospital de Clínicas de Porto Alegre, Brazil (June 28, 2010 - project number 100024).

## Results

In our sample of 302 patients, age ranged from 12 to 87 years (mean 49±16 years old) (p = 0.98). 123 patients (40.7%) were premenopausal and 130 (43.05%) were postmenopausal (p = 0.97). The mean parity was 2.50±1.38 children. Regarding symptoms, 159 patients (52.7%) reported lower abdominal pain, whereas 68 patients (22.5%) did not (p = 0.08). On physical examination, 152 patients (50.3%) had a palpable mass and 61 (20.2%) did not (p < 0.001). Table 1 shows overall patient characteristics, information regarding missing information on each variable, and distribution of patients according to sonographic morphology and size of the mass (≤10 cm or >10 cm). 121 patients (40.1%) had infraumbilical masses, whereas 23 (7.6%) had masses extending infra and supraumbilical (p = 0.001). The surgical approach was open in 220 patients (72.8%) and laparoscopic in 77 (25.5%); (p < 0.001). Intraoperative capsule rupture occurred in 35 patients (11.6%) and did not occur in 258 (85.4%) (p = 0.051). 9 patients (3.0%) had a family history of breast cancer, whereas 176 (58.3%) did not. 6 patients (2.0%) had a family history of ovarian neoplasms, while 144 (47.7%) did not. 140 patients (46.4%) had a history of surgical procedures, whereas 84 (27.8%) did not. Serum CA-125 levels were <35 IU/mL in 183 patients (60.6%) and >35 IU/mL in 93 (30.8%), as shown in table 1.

**Table 1:** Distribution of patients according to sonographic morphology and size of adnexal mass

Variables	Group (tumor ≤10 cm^a^)				Group (tumor >10 cm^a^)				Overall n = 302	Patients missing data %	p-value
	Unilocular (homogeneous) lesions	Septated cystic lesions	Heterogeneous (solid/cystic) lesions	Solid lesion	Unilocular (homogeneous) lesions	Septated cystic lesions	Heterogeneous (solid/cystic) lesions	Solid lesion			
	n = 33	n = 32	n = 90	n = 24	n = 15	n = 39	n = 63	n = 6			
Age (years)	47±12	49±16	50±16	49±18	50±19	47±14	49±17	47±6	49±16	0	0.98
Menopausal status										16.2	0.97
Pre-menopausal	15	14	35	10	5	15	27	2	123		
Post-menopausal	12	15	44	10	9	13	25	2	130		
Parity (no. of offspring)	2±1.5	2±1.8	2±2	2±2.7	2±1.5	3±2.6	2±2.3	2.5±1.4	2.4±2.2	19.9	N/C
Lower abdominal pain	16	14	42	11	8	25	41	2	159	24.8	0.08
Family history of breast cancer	2	2	4	0	0	0	1	0	9	38.7	N/C
Family history of ovarian cancer	0	1	2	0	0	1	2	0	6	50.3	N/C
Past surgical history	19	18	43	8	6	17	26	3	140	25.8	N/C
Palpable mass	11	15	32	13	10	23	44	4	152	29.5	<0.001
Site										52.3	0.001
Infraumbilical	9	13	32	13	7	15	28	4	121		
Infra/supraumbilical	0	0	2	0	1	7	13	0	23		
CA-125										8.6	<0.001
≤35 UI/mL	1	23	63	19	12	25	18	2	183		
>35 UI/mL	6	8	20	4	3	10	39	3	93		
Surgical approach										1.7	<0.001
Laparotomy	17	22	49	16	13	35	62	6	220		
Laparoscopic	16	9	39	7	2	3	1	0	77		
Capsule rupture	1	7	5	2	2	5	13	0	35	3.0	0.051
IFS											
Benign tumors	31(93.9)	31(96.7)	80(88.9)	20(83.3)	15(100)	34(87.2)	39(62)	5(83.3)	255(84.4)		
Borderline tumors	1(3)	0(0)	1(1.1)	1(4.2)	0(0)	4(10.7)	8(12.7)	0(0)	15(5)		
Malignant tumors	1(3)	1(3.1)	9(10)	3(12.5)	0(0)	1(2.6)	16(25.4)	1(16.7)	32(10.6)		
HPE											
Benign tumors	31(94)	31(97)	78(86.7)	19(79.2)	14(93.3)	33(84.6)	34(54)	5(83.3)	245(81.1)		
Borderline tumors	1(3)	0(0)	2(2.2)	1(4.2)	0(0)	4(10.3)	9(14.3)	0(0)	17(5.6)		
Malignant tumors	1(3)	1(3.1)	10(11.1)	4(16.7)	1(6.7)	2(5.1)	20(31.8)	1(16.7)	40(13.3)		

IFS - Intraoperative Frozen Section; HPE - Histopathology Examination; N/C - not calculated

### Overall agreement between IFS and HPE in 302 patients

In IFS, 255 masses were classified as benign (84.4%), 15 as borderline (5.0%), and 32 as malignant (10.6%). In HPE, 245 cases were diagnosed as benign (81.1%), 17 as borderline (5.6%), and 40 as malignant (13.3%). The agreement between IFS and HPE was as follows: 96.1% for benign tumors, 96.1% for malignant tumors, and 73.3% for borderline tumors (Table 2). The age groups with the lowest agreement were 70 to 79 years (87%) and highest agreement was 80 to 89 years (100%).

**Table 2:** Diagnostic agreement between IFS and HPE according to study group (combined morphology and size)

Group	Agreement n(%)	Kappa
Unilocular ≤10 cm	33(100)	1.000
Septated/cystic ≤10 cm	32(100)	1.000
Heterogeneous–solid/cystic ≤10 cm	90(97.8)	0.898
Solid ≤10 cm	24(95.8)	0.869
Unilocular >10 cm	15(93.3)	N/C
Septated/cystic >10 cm	39(89.7)	0.591
Heterogeneous–solid/cystic >10 cm	63(87.3)	0.776
Solid >10 cm	6(100)	1.000
Benign tumors	96.1%	N/C
Borderline tumors	96.1%	N/C
Malignant tumors	73.3%	N/C

N/C - not calculated

### Agreement between IFS and HPE according to morphology and tumor size

Table 2 shows the agreement between the diagnosis made with IFS and HPE according to the study group, i.e., combining size and morphology. In group 1 (33 cases, unilocular lesions ≤10 cm), group 2 (32 cases, septated cystic lesions ≤10 cm), and group 8 (6 cases, solid lesions >10 cm) there was 100% agreement between IFS and HPE, with final diagnoses of 67 benign (94.4%), 1 borderline (1.4%), and 3 malignant tumors (4.2%). In Group 3 (90 cases, heterogeneous lesions ≤10 cm), there were 2 divergences (2,2%): 1 case (1.1%) classified as benign in IFS and as borderline in HPE, and 1 case (1.1%) classified as benign in IFS and found to be malignant in HPE. In Group 4 (24 cases, solid lesions ≤10 cm), there was 1 divergence (4.2%) – a mass considered benign in IFS and found to be malignant in HPE. In Group 5 (15 cases, unilocular lesions >10 cm), there was 1 divergence (7.1%), a mass considered benign in IFS and found to be malignant in HPE. In Group 6 (39 cases, septated cystic lesions >10 cm), 4 divergences (10.3%) were detected, with 2 (50%) masses classified as benign in IFS and as borderline in HPE, 1 (25%) classified as borderline in IFS and benign in HPE, and 1 (25%) classified as borderline in IFS and malignant in HPE (p < 0.001). In Group 7 (63 cases, heterogeneous lesions >10 cm), there were 8 divergences (12.7%): 3 (37.5%) were masses classified as benign in IFS and borderline in HPE, 3 (37.5%) were benign in IFS and malignant in HPE, 1 (12.5%) was borderline in IFS and benign in HPE, and 1 (12.5%) was borderline in IFS and malignant in HPE.

The histological types that had the greatest agreement comparing the frozen section with the final pathology are dermoid cyst, ovarian fibroma, and mucinous cystadenoma. And the histological types that had the least agreement are endometrioid adenocarcinoma, serous adenocarcinoma, and mucinous cystadenocarcinoma borderline.

## Discussion

The present study set out to identify a subgroup of patients with adnexal masses in whom we should wait for definitive HPE for the staging decision despite a negative IFS. In this study we compared IFS with HPE as diagnostic gold standard. Considering a combination of tumor size and morphology, we observed perfect agreement between IFS and HPE for unilocular and septated/cystic masses ≤10 cm and for solid masses >10 cm. Disagreements were observed for all other combinations, suggesting that staging surgery for ovarian cancer should be based on final HPE for heterogeneous tumors regardless of size, for solid tumors smaller than 10 cm, and for all non-solid tumors larger than 10 cm. Despite the small sample size, the present results provide an interesting insight, which deserves to be further explored.

As a more general rule, it seems wise to wait for final pathology results before staging tumors classified as benign and especially borderline in IFS; misclassification of malignant tumors as benign is a critical event, with a major impact on prognosis.^(12-14)^The accuracy of IFS classification has been extensively analyzed.^(5,7,9,15-20)^ The divergence between IFS and HPE has been ascribed to sampling error, misinterpretation, pathologist inexperience, breakdowns in communication between surgeon and pathologist, and technical issues.^(1,9,15,19,21)^ IFS results do not depend only on microscopy, but they are also related to clinical hypothesis and macroscopic assessment of the surgical specimen.^(3,22,23)^ In one study, gross pathology criteria were able to distinguish benign lesions from malignant lesions with 93% sensitivity.^(19)^Nevertheless, the fact the IFS may prevent the need for additional surgery in women with adnexal masses, the identification of specific criteria associated with IFS success is desirable.

In that sense, it is interesting that agreement of 100% between IFS and HPE was obtained in the present study only for the combination of morphologic findings and tumor size. A similar finding has been reported by Rogers et al.^(10)^in a study including 126 pediatric and adolescent patients who underwent operative management of adnexal masses, a combination of ultrasound finding of tumor size ≥8 cm and tumor complexity identified 100% of the malignancies. In turn, 36% of benign tumors were both ≥ 8 cm in size and complex (p < 0.001). Along with the present results, this supports the evidence that a combination of features might enable optimal preoperative assessment, and also help with accurate IFS diagnoses.

High agreement between IFS and HPE for cystic tumors ≤10 cm in size has been previously reported.^(12,24,25)^In another study of 286 patients, 184 ovarian tumors e 102 uterine tumors, the sensitivity and specificity of IFS for ovarian neoplasms benign, borderline, and malignant tumors were 100%, 66.7%, 96.9% and 97.1%, 99.4%, 100% respectively.^(9)^Thus, this study showed that IFS can contribute significantly to determining the malignant or benign nature of ovarian epithelial tumors, whereas for borderline tumors its accuracy seems to be more dependent on the pathologist’s experience and tumor.^(9)^ In the study by Brun et al. (2008),^(2)^ borderline tumors misdiagnosed as benign in frozen section were more likely to be small, unilocular, fluid, mucinous, or exhibit small foci of atypia (less than 10% of the total sample), thus increasing sampling error and interpretation. As suggested by the present results, tumors identified as borderline in IFS should always undergo staging after HPE for safe diagnosis. This recommendation is also supported by a study of borderline tumors in 120 patients, which reported a diagnostic disagreement rate of 13.3%: 15 patients (12.5%) who were diagnosed as borderline in IFS were reclassified as malignant after final histopathology, and 1 (0.8%) tumor originally diagnosed as borderline in IFS was deemed benign in HPE.^(26)^ That study also found that the risk of underdiagnosis due to IFS limitation is greatest for tumors larger than 8 cm (p = 0.004).^(26)^Ovarian adnexal masses are graded based on the International Ovarian Tumor Analysis (IOTA) criteria. The classification of the IOTA criteria is based on various features observed on imaging studies, such as transvaginal ultrasound. The criteria include aspects such as: (i) echo patterns, (ii) margins, (iii) presence of septa and projections, (iv) vascularization, and (v) morphologic characteristics. These criteria are used to differentiate benign from malignant tumors and to estimate the likelihood of malignancy. Consequently, the IOTA criteria have demonstrated effectiveness in discerning benign from malignant tumors, helping to prevent unnecessary surgery for benign tumors and facilitating prompt intervention in cases of malignancy. It is important to remember that although the IOTA criteria are useful in the initial evaluation of ovarian tumors, the final diagnosis of malignancy is usually confirmed by biopsy or pathologic analysis after surgical removal of the tumor.^(27,28)^

We found an agreement of 100% between IFS and HPE for the diagnosis of solid tumors >10 cm in size. This was contrary to our expectation, as the literature reports reductions in IFS sensitivity with increasing tumor size.^(18)^Some studies have found tumor size to be the foremost predictor of diagnostic failure of IFS, with decreased accuracy for tumors larger than 10 cm or 20 cm.^(4,24)^ A retrospective study also concluded that, especially in large masses, high precision while sampling representative tissue for the frozen section and the cooperation of surgeon and pathologist can increase the value of this method.^(17)^Thus, it seems that the combination of size with morphology as proposed in the present study should be considered for the decision regarding IFS accuracy.

Some limitations of this study must be mentioned, including its retrospective design. Surgical specimens were examined by different pathologists with varying degrees of experience; pathologists who examined frozen sections were not always those who performed the final HPE conventional examination. Pelvic ultrasounds were performed in different departments by radiologists with varying degrees of experience, and time between pelvic ultrasound and surgery was not standardized. In addition, clinical information was lacking for some variables. Nevertheless, the study provides initial evidence of the usefulness of combining criteria tumor size and morphology to assess the accuracy of IFS.

## Conclusion

In conclusion, the stratification of adnexal masses according to size and morphology is a good method for preoperative assessment. We conclude that a staging decision should wait for final pathology report, especially in heterogeneous adnexal tumors of any size, for solid tumors smaller than 10 cm, and for all non-solid tumors larger than 10 cm. In addition, also in borderline tumors and in young patients who desire fertility. In these situations, we suggest waiting for final report instead of a radical procedure based on frozen section or maybe do not indicate IFS in these situations.
